# Is early TMJ involvement in children with juvenile idiopathic arthritis clinically detectable? Clinical examination of the TMJ in comparison with contrast enhanced MRI in patients with juvenile idiopathic arthritis

**DOI:** 10.1186/s12969-015-0056-2

**Published:** 2015-12-09

**Authors:** Heidi Keller, Lukas Markus Müller, Goran Markic, Thomas Schraner, Christian Johannes Kellenberger, Rotraud Katharina Saurenmann

**Affiliations:** Clinic for Orthodontics and Pediatric Dentistry, University of Zürich, Zürich, Switzerland; Department of Diagnostic Imaging, University Children’s Hospital Zürich, Zürich, Switzerland; Children’s Research Center, University of Zürich, Zürich, Switzerland; Division or Rheumatology, University Children’s Hospital Zürich, Zürich, Switzerland

**Keywords:** Juvenile idiopathic arthritis, Temporomandibular joint arthritis, Diagnosis, Magnetic resonance imaging, Maximal mouth opening capacity

## Abstract

**Background:**

To test clinical findings associated with early temporomandibular joint (TMJ) arthritis in comparison to the current gold standard contrast enhanced magnetic resonance imaging (MRI) in children with juvenile idiopathic arthritis (JIA).

**Methods:**

Seventy-six consecutive JIA patients were included in this study. Rheumatological and orthodontic examinations were performed blinded to MRI findings. Joint effusion and/or increased contrast enhancement of synovium or bone as well as TMJ deformity were assessed on MRI and compared to clinical findings. The maximal mouth opening capacity (MOC) of the JIA patients was compared to normative values obtained from a cohort of 20719 school children from Zürich, Switzerland.

**Results:**

On MRI a total of 54/76 (71 %) patients and 92/152 (61 %) joints had signs of TMJ involvement. MRI showed enhancement in 85/152 (56 %) and deformity in 39/152 (26 %) joints. MOC, asymmetry and restriction in condylar translation showed significant correlation to TMJ enhancement and deformity, whereas antegonial notching was correlated with TMJ deformity only. When joints with deformity were excluded, enhancement alone did not show a significant correlation with any clinical factor.

**Conclusions:**

Clinical findings in affected TMJs are correlated with structural damage only. Therefore clinical assessment of TMJs does not allow to diagnose early arthritis accurately and will still depend on contrast enhanced MRI.

## Background

Juvenile idiopathic arthritis (JIA) is the most common rheumatic disease during childhood and adolescence with a prevalence of 0.07 to 4.01 per 1000 children depending on ethnical and geographical factors [1]. Involvement of temporomandibular joints (TMJ) varies highly depending on the diagnostic methods used, the population and JIA subgroups studied, ranging from 17 % to 87 % [2–6]. Contrast enhanced magnetic resonance imaging (MRI) is considered the gold standard to reliably diagnose both acute TMJ arthritis and deformation of the TMJ [3, 7, 8] and may have direct impact on treatment decisions [9].

The main growth centre of the mandible is located in the condyle separated from the joint space only by a thin layer of fibrocartilage. This makes mandibular growth vulnerable to arthritic changes of the TMJ [10] eventually resulting in mandibular retrognathia, posterior rotation or facial asymmetry in cases with unilateral TMJ involvement [11–13]. In the course of JIA the TMJ can be the first or even the only joint affected [14, 15]. Unfortunately detection of TMJ arthritis in children with JIA is difficult as early signs and symptoms are missing in most patients. Therefore, diagnosis of TMJ involvement is often late when growth disturbances are already obvious. In a study of Weiss et al. [8] 71 % of the patients with acute arthritis diagnosed with MRI were asymptomatic. Twilt et al. [5] observed that only 12 % of the patients with signs of TMJ involvement on orthopantogram reported pain and in only 5 % a swelling was detectable. Other studies reported an even lower prevalence of symptoms in case of TMJ arthritis [16, 17]. Since MRI is not available everywhere and the imaging procedure especially for young children is demanding, other examination methods would be desirable to detect early onset of TMJ involvement in children with JIA. The maximal mouth opening capacity (MOC) is probably the most common factor analysed in relation to TMJ involvement. A correlation between reduced MOC and TMJ arthritis is reported by several studies [2, 16, 18–20], while others did not find a clear association [5, 21]. Moreover it is difficult to quantify the effect of TMJ involvement on the MOC in children with JIA as age matched controls are often missing in the literature.

Other clinical parameters associated with TMJ arthritis are deviation of mouth opening, TMJ crepitation, mandibular asymmetry or retrognathia [5, 18, 19, 22].

The aim of this study was to test clinical findings associated with early TMJ arthritis in relation to the current gold standard contrast enhanced MRI.

## Methods

### Patients

Between March 2006 and October 2008 83 consecutive patients with a diagnosis of JIA according to the ILAR 2003 criteria [23] were included in this study after informed consent was obtained. Exclusion criteria were a previous diagnosis of TMJ arthritis, performance of MRI of the TMJs within the last 6 months and orthodontic treatment within the past 12 months. Date of birth, sex, subtype of JIA, disease onset date, date of diagnosis and medication up to the study entry were extracted from the patient’s hospital chart. The study was approved by the Institutional and Governmental Ethics Review Board.

### Methods

Patients underwent three different examinations performed by different medical and dental specialists:

I. MRI of the TMJs reviewed by two paediatric radiologists
II. Rheumatological examination by a staff paediatric rheumatologist
III. Orthodontic examination by an orthodontist

For each patient the clinical examinations were scheduled within a time frame of less than 3 months from MRI. All examiners were blinded to the results of the other examination methods.

#### Magnetic resonance imaging

Both TMJs were imaged on a 1.5 Tesla system (Signa MR/iTwinspeed scanner, GE Medical Systems, Milwaukee, Wisconsin, USA) with a dedicated TMJ coil in closed mouth position. Axial and coronal T2-weighted fast spin echo localizers were acquired for adjusting the subsequent sagittal oblique sequences perpendicular to each mandibular condyle and parallel to each mandibular ramus. Sagittal oblique images were acquired with a 2 mm slice thickness, without gap, 12 cm field of view and 256 x 224 matrix. First T1-weighted spoiled gradient echo images (flip angle 80°, TR 325 ms, TE 4.2 ms), proton density fast spin echo images (TR 2660 ms, TE 25 ms, ETL 8) and fat-saturated T2-weighted fast spin echo images (TR 2840 ms, TE 86 ms, ETL 16) were performed, followed by contrast-enhanced fat-saturated T1-weighted fast spin echo images (TR 600 ms, TE 11 ms, ETL 3) acquired within 5 min after intravenous administration of a single dose (0.1 mmol/kg bodyweight) of gadolinium based contrast medium (dimegluminegadopentate, Magnevist, Bayer AG, Switzerland; or gadodiamide, Omniscan, GE Healthcare AG, Switzerland).

Children who were not able to lie sufficiently still for the duration of the MRI had the examination performed under propofol sedation (*n* = 23/76) applied by anaesthesiologists according to hospital routine.

All MRI studies were reviewed by two independent radiologists. Differences in interpretation were discussed until consensus was reached. Each TMJ was assessed for the presence of an effusion, increased contrast enhancement and deformity of the mandibular condyle. On T2-weighted images, small dots or lines of high signal in a joint recess without distension were considered a normal amount of joint fluid. On fat-saturated T1-weighted images, high signal confined to intraarticular fluid as delineated on the T2-weighted images was considered normal joint enhancement [24]. Increased joint enhancement was graded as mild, when the signal of the synovial membrane was hyperintense to muscle and as severe when the synovial membrane was thickened with signal isointense to vessels. Deformity of the mandibular condyle was graded as mild when only the anterior or posterior circumference was flattened, and as severe when the condyle was squared with loss of height [25]. Presence of a joint effusion and/or increased enhancement was considered indicative for active inflammation.

#### Rheumatologic examination

The rheumatologic evaluation included examination of all joints for signs of inflammation such as swelling, tenderness and limitation of range of motion, especially restriction in condylar translation. The TMJ was palpated with and without movement of the jaw, while pain at the joint space or at the condylar head was registered. MOC, i.e. the unassisted maximal interincisal distance without correction for overbite, was measured with an acrylic ruler after the patient had been asked to open the mouth as wide as possible several times for warm up. The jawline was visually assessed for mandibular asymmetry or retrognathism both known as signs for condylar growth disturbances.

#### Orthodontic examination

The orthodontic examination included a detailed questionnaire about TMJ function, pain and symptoms.

TMJ palpation, compression and distraction tests were performed and TMJ noise was recorded. TMJ pain and tenderness of masticatory muscles (Mm. masseter and temporalis) were recorded using the “Oucher Scale” (range of pain intensity 0–10), validated for children (between 3 to 12 years of age) [26], or on a VAS (0–10 cm) for children older than 12 years.

The highest value of three MOC exercises without correction for overbite was registered.

The morphology of the lower jaw was visually and manually assessed with regard to mandibular retrognathism, asymmetry and palpable antegonial notching. Mild asymmetries and mandibular retrognathism were considered normal variations of facial morphology.

The measured MOC values from both the rheumatologic and orthodontic examinations were compared to normative values obtained from a cohort of 20’719 swiss school children [27], with an age range from 3–18 years. A standard deviation score (SDS) and the corresponding centile for each individual measurement of every JIA patient were calculated applying the LMS method [28].

#### Evaluation and statistical analysis

The MRI results were considered the gold standard or “true” value against which the rheumatologic and orthodontic examination were compared. Either MRI signs of active inflammation (effusion, increased enhancement) or presence of TMJ deformity were considered TMJ involvement. If the orthodontic or rheumatological examination took place before MRI, the time interval was recorded as negative.

The different examination methods and parameters were tested for their reliability to pre-estimate presence or absence of active arthritis, TMJ deformity and TMJ involvement.

Standard statistical software packages SPSS version 20.0.0 (Chicago, Ill, USA) were used for statistical analysis.

Shapiro-Wilk and Kolmogorov-Smirnov tests were applied to check normality assumptions.

Differences in between MOCs and their corresponding SDS and centiles and MRI findings were assessed using one-way ANOVA with Bonferroni post-hoc correction. Associations between the MRI involvement and categorical factors were analysed using the Chi-square test.

Results of statistical analysis with p-values smaller than 0.05 were considered statistically significant.

## Results

From 83 patients enrolled in the study, 7 patients were excluded: 2 patients because the interval between MRI and clinical examinations exceeded 3 months. 3 patients were not able to lie sufficiently still towards the end of MRI resulting in non-diagnostic contrast-enhanced images. In one patient, who had MRI performed under anaesthesia, the inflamed TMJ was injected with corticosteroids on the same occasion and before the orthodontic examination had taken place. One patient decided to discontinue the study after MRI was performed.

Therefore, 76 patients were available for comparison of the orthodontic examination to MRI. The mean time interval between MRI and the orthodontic or rheumatological examination was −5.03 days (median −1, SD 20.2, range (−84,64)) and −23.0 days (median −24, SD 30.5; range (−88,52)) respectively. For one patient data of the rheumatologic examination was missing, therefore only 75 patients were analysed. Furthermore there was one MOC value missing from the rheumatologic examination. Four patients were younger than 3 years of age, therefore their SDS values and the centiles could not be calculated. For the characteristics of the 76 patients regarding JIA subtype, disease duration, disease activity and treatment see Table 1.

**Table 1:** Patients characteristics

Patients characteristics	All patients	Patients with TMJ involvement on MRI	Patients without TMJ involvement on MRI
Total no. of patients, n (%)	76	53 (70)	23 (30)
Female, n (%)	42 (55)	29 (69)	13 (31)
Oligoarticular, n (%)	24 (32)	17 (32)	7 (30)
Enthesitis related arthritis n (%)	9 (12)	7 (13)	2 (9)
Oligoarticular extended, n (%)	11(15)	7 (13)	4 (17)
Polyarticular RF neg, n (%)	25 (33)	19 (36)	6 (26)
Systemic, n (%)	1 (1)	1 (2)	0 (0)
PsA, n (%)	2 (3)	1 (2)	1 (4)
Unclassified, n (%)	4 (5)	1 (2)	3 (13)
Age at diagnosis, median (range), years	5.5 (1–14.9)	5.4 (1–19.4)	6.6 (1.1–14.1)
Age at examination, median (range), years	9.7 (1.9–18.6)	9.6 (1.9–18.6)	9.8 (4.8–17.8)
Disease duration, median (range), years	2.4 (0.0–15.7)	1.9 (0.0–15.4)	3.5 (0.1–15.7)
HLA-B27 positive/tested, n (%) (*n* = 59)	6 (10)	4 (10)	2 (11)
ANA positive, n (%) (*n* = 74)	47 (64)	33 (62)	14 (61)
Uveitis, n (%)	11 (15)	6 (11)	5 (22)
Treatment with systemic disease-modifying drugs, n (%)	31 (41)	23 (43)	8 (35)
Infliximab, n (%)	4 (5)	4 (10)	0 (0)
Etanercept, n (%)	1 (1)	0 (0)	1 (4)
Methotrexate, n (%)	31 (41)	23 (43)	8 (35)

TMJ involvement on MRI = signs of inflammation (effusion/increased enhancement) and/or deformation

### Magnetic resonance imaging

TMJ involvement was present on MRI in 54/76 patients (71 %) and 92/152 joints (61 %). Signs of active TMJ arthritis were present in 52/76 patients (68 %) and 85/152 joints (56 %). Arthritis was unilateral in 16/54 patients (30 %). Enhancement was mild in 67 and severe in 18 joints. An increased amount of joint fluid was found in 11 TMJs of 10 patients. In 2 TMJs of the same patient an increased amount of joint fluid was the only sign of inflammation. Condylar deformity was present in 25/76 patients (33 %) and 39/152 joints (26 %) and was mild in 15 and severe in 24 joints. All 18 TMJs with severe enhancement had condylar deformities of which 15 were severe. 14 of 67 TMJs with mild contrast enhancement had condylar deformities which were mild in 10 cases. In seven joints with deformity, out of which five were severe, no enhancement was detectable. The correlation between the degree of enhancement and the degree of condylar deformity was statistically significant (Chi-square *p* < 0.0001 for the difference between the groups).

### Rheumatologic examination

22/75 (29 %) patients reported pain during rheumatologic TMJ examination out of which 12 patients complained about bilateral pain. The mean MOC registered during the examination was 44,8 mm (range 32–59), the mean SD-Score −0,05 and the mean centile 47,4. 12/70 (17 %) and 26/70 (37 %) patients revealed a MOC smaller than the 10^th^ and 25^th^ percentile respectively. Of the 12 patients with an MOC smaller than the 10^th^ percentile, MRI showed enhancement in 9 and deformity in 6. In three patients with MOC smaller than the 10^th^ percentile no TMJ involvement was visible. Condylar growth disturbance was diagnosed in 34/75 (45 %) patients. 12/75 (16 %) patients showed a mandibular retrognathism and 28/75 (37 %) a mandibular asymmetry.

### Orthodontic examination

The assessment by questionnaire in terms of pain was positive in 28/76 (37 %) patients. The mean pain level reported was 3,3 (range 0–7).

The clinical examination of the TMJ and muscles (M. masseter and M. temporalis) was painful in 35/76 (46 %) and 32/68 (47 %) patients respectively. Muscle examination was not performed in 8 patients due to limited cooperation because of young age.

TMJ clicking and crepitation in was present in 15/76 (20 %) and 16/76 (21 %) patients respectively.

The mean MOC was 44,6 mm (range 29–59), the mean SD-Score −0,07 and the mean centile 49,4. 13/72 (18 %) and 20/72 (28 %) patients revealed a MOC smaller than the 10^th^ and 25^th^ percentile respectively. Out of the patients with MOC smaller than the 10^th^ percentile 10 showed enhancement and 7 deformity on the MRI.

Palpable antegonial notching was found in 41/76 (54 %) patients with bilateral findings in 26/76 (34 %) cases. 13/76 (17 %) patients showed severe mandibular retrognathism and 31/76 (41 %) severe mandibular asymmetry.

### Clinical parameters associated with TMJ involvement in MRI

**Fig. 1:**
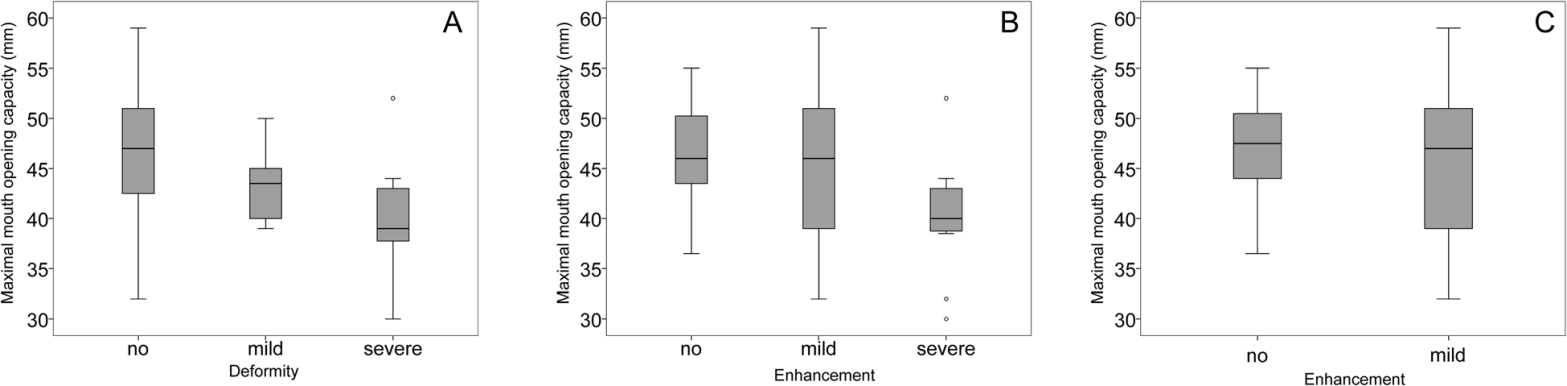
Boxplots of MRI vs. maximal mouth opening capacity. **a** Deformity vs. maximal mouth opening capacity (orthodontic examination, *n* = 76). **b** Enhancement vs. maximal mouth opening capacity (orthodontic examination, *n* = 76). **c** Enhancement (excluding patients with deformity) vs. maximal mouth opening capacity (orthodontic examination, *n* = 51)

Absolute values, SD-scores and centiles of MOC assessed by the orthodontic as well as the rheumatologic examination showed significant correlation to TMJ involvement on MRI (Table 2/Table 3). Subgroups with no deformity and severe deformity showed highly significant differences in MOC, (Table 2/Fig. 1a). Subgroups with no, mild and severe enhancement did not always correlate with MOC (Table 3, Fig. 1b). When excluding all the patients with deformity on the MRI, 29 patients with mild and 22 patients with no enhancement showed no significant correlation with the MOCs, the SD-Scores and the centiles (Fig. 1c/Table 4).

**Table 2:** Analysis of MRI deformity vs. MOC, corresponding SDS-values and centiles

		MRI deformity					
Factor	Examination method	ANOVA	Bonferroni group comparison				
		p-value	Severity subgroups		p-value	Mean difference (S1 - S2)	CI95 (lower, upper)
			S1	S2			
Maximal mouth opening capacity	Orthodontic (*n* = 76)		0	1	0.467	3.0	−2.1, 8.1
		0.001*	0	2	0.001*	6.9	2.5, 11.1
			1	2	0.378	3.8	−2.2, 9.8
	Rheumatologic (*n* = 74)		0	1	0.076	4.7	−0.3, 9.7
		0.002*	0	2	0.004*	6.0	1.6, 10.4
			1	2	1.000	1.3	−4.7, 7.4
SDS values	Orthodontic (*n* = 72)		0	1	0.312	0.6	−0.3, 1.5
		<0.001*	0	2	<0.001*	1.6	0.8, 2.4
			1	2	0.760	1.0	−0.1, 2.0
	Rheumatologic (*n* = 70)		0	1	0.071	0.9	−0.1, 1.9
		<0.001*	0	2	<0.001*	1.5	0.6, 2.3
			1	2	0.769	0.5	−0.6, 1.7
Centiles	Orthodontic (*n* = 72)		0	1	0.142	19.5	−4.2, 43.3
		<0.001*	0	2	<0.001*	42.3	22.0, 62.5
			1	2	0.147	22.7	−5.1, 50.5
	Rheumatologic (*n* = 70)		0	1	0.023*	26.9	2.8, 50.9
		<0.001*	0	2	<0.001*	42.5	21.4, 63.5
			1	2	0.552	15.6	−12.9, 44.2

One way Anova with post-hoc Bonferroni test to compare the MRI to the MOC’s and the corresponding SDS-values and centiles. All continuous variables were normally distributed. Mean difference between groups with upper and lower 95 % confidence interval (CI95) is provided

MRI deformity subgroups: 0 = normal, 1 = mild, 2 = severe

*p-value < 0.05

**Table 3:** Analysis of MRI enhancement vs. MOC, corresponding SDS-values and centiles

		MRI enhancement					
Factor	Examination method	ANOVA	Bonferroni group comparison				
		p-value	Severity subgroups		p-value	Mean difference (S1 - S2)	CI95 (lower, upper)
			S1	S2			
Maximal mouth opening capacity	Orthodontic (*n* = 76)		0	1	1.000	1.4	−2.6, 5.4
		0.005*	0	2	0.005*	6.6	1.7, 11.6
			1	2	0.022*	5.2	0.6, 9.8
	Rheumatologic (*n* = 74)		0	1	1.000	0.5	−3.5, 4.5
		0.048*	0	2	0.064	4.9	−0.2, −0.4
			1	2	0.081	4.4	−0.4,9.8
SDS values	Orthodontic (*n* = 72)		0	1	1.000	<−0.1	−0.7, 0.7
		<0.001*	0	2	0.001*	1.4	0.5, 2.3
			1	2	0.001*	1.4	0.5, 2.2
	Rheumatologic (*n* = 70)		0	1	1.000	−0.1	−0.9, 0.7
		0.008*	0	2	0.027*	1.1	<0.1, 2.1
			1	2	0.008*	1.2	0.2, 2.2
Centiles	Orthodontic (*n* = 72)		0	1	1.000	−0.3	−19.6, 19.1
		<0.001*	0	2	0.001*	35.4	11.7, 59.1
			1	2	0.001*	35.7	13.3, 58.1
	Rheumatologic (*n* = 70)		0	1	1.000	−1.7	−21.9, 18.4
		0.002*	0	2	0.060	33.0	8.0, 58.1
			1	2	0.002*	34.8	10.9, 58.6

One way Anova with post-hoc Bonferroni test to compare the MRI to the MOC’s and the corresponding SDS-values and centiles. All continuous variables were normally distributed. Mean difference between groups with upper and lower 95 % confidence interval (CI95) is provided

MRI enhancement subgroups: 0 = normal, 1 = mild, 2 = severe

*p-value < 0.05

**Table 4:** Analysis of MRI findings after excluding deformity vs. MOC, corresponding SDS-values and centiles

MRI enhancement without deformity			
Factor	Examination method	*T*-test p-value	Mean difference (CI95 lower, CI95 upper)
Maximal mouth opening capacity	Orthodontic (*n* = 51)	0.506	1.2 (−2.4, 4.7)
	Rheumatologic (*n* = 50)	0.719	−0.7 (−4.4, 3.0)
SDS values	Orthodontic (*n* = 47)	0.698	−0.1 (−0.8, 0.5)
	Rheumatologic (*n* = 46)	0.265	−0.4 (−1.2, 0.3)
Centiles	Orthodontic (*n* = 47)	0.683	−3.6 (−21.0, 13.9)
	Rheumatologic (*n* = 46)	0.270	−10.5 (−29.5, 8.5)

*T*-test to compare the MRI findings to the MOC’s and the corresponding SDS-values and centiles after excluding patients with deformity. Mean difference between groups with upper and lower 95 % confidence interval (CI95) is provided. All continuous variables were normally distributed

*p-value < 0.05

We did not find significant correlation of any record of pain with active TMJ arthritis, neither in the questionnaire, nor in the clinical orthodontic or rheumatologic examination. Correlation between MRI findings and clinical parameters such as asymmetry, retrognathia, restriction in condylar translation, antegonial notching and TMJ noises are display in Table 5.

**Table 5:** Analysis of MRI findings vs. clinical parameters

	Pain		Asymmetry		Retrognathia		Restriction in condylar translation	Antegonial notching	Clicking/crepitation
	Orthodontic examination	Rheumatological examination	Orthodontic examination	Rheumatological examination	Orthodontic examination	Rheumatological examination	Rheumatological examination	Orthodontic examination	Orthodontic examination
MRI enhancement (*n* = 76)	0.662	0.070	0.002*	0.198	0.308	0.448	0.020*	0.067	0.866
MRI deformation (*n* = 76)	0.600	0.669	<0.001*	0.072	0.085	0.430	0.001*	0.014*	0.701
MRI enhancement only (*n* = 51)	0.964	0.213	0.906	0.231	0.259	0.948	0.371	0.449	0.782

Chi-square test to compare the MRI findings to clinical parameters assessed during the orthodontic or rheumatological examination (p-values)

*p-value < 0.05, NS = not significant

## Discussion

In this study history and clinical findings of 76 patients collected by different specialists were compared to the diagnostic gold standard contrast enhanced MRI. This study is unique because of centiles used to quantify and compare MOC between the groups.

### Contrast—enhanced MRI

As mentioned before it is of utmost importance to distinguish between early signs of inflammation and later occurring structural damage. Therefore, the MRI findings were carefully graded into different stages of inflammation and deformity by two experienced paediatric radiologists. The high frequency of TMJ involvement in our sample (71 %) is in line with previous studies [2, 5, 29] with MRI based diagnosis [8, 19, 30, 31]. We found a higher than expected frequency of TMJ involvement in patients with oligoarticular arthritis (70 %) or enthesitis related arthritis (77 %) than previous studies [6] which is likely due to our small study sample. In contrast to our study with 68 % of the patients showing active arthritis, Stoll *et al*. [18] with comparable diagnostic criteria revealed only 36 %, whereas Weiss et al. [8] showed as much as 75 %. The latter study however applied a different definition of active TMJ arthritis, namely assessing effusion or synovial thickening instead of enhancement. Condylar deformity was observed in 33 % of our patients, which is in accordance with some studies [16, 31] while others reported higher rates [8] with numbers increasing over time [17].

### Rheumatological and orthodontic examination

Pain assessed by a detailed questionnaire was present in 37 % in our study even if the mean reported pain level was low. The frequency of reported TMJ pain in JIA patients is usually low [8, 32], even in cases with a severe condylar deformity [33]. Twilt et al. [5] showed that only 11 out of 97 children had a history of pain, suggesting that pain is neither a sensitive nor a specific tool for detecting TMJ synovitis or deformation. A reason for the relatively high rate in our study could be the very detailed questionnaire given to the patients and their parents before the examination allowing them to reflect on distinct situations in their daily routine.

TMJ pain during examination was also more frequent than usual in our cohort with 29 % with muscle pain during the rheumatologic and 46 % during the orthodontist examination. Findings from several studies regarding joint and muscular pain during examination range from 7 % up to 35 % [8, 34, 35]. When we compared our numbers to the frequency of temporomandibular disorders (TMD) of healthy adolescents, we found that they are within the frequency range of TMD in this age group [35–37]. Although our cohort is younger, one should therefore bear in mind the possibility of an overlap between symptoms of arthritis and a diagnosis of TMD. As expected from other reports [4, 27, 33], also in our study pain was neither a sensitive marker for early TMJ arthritis nor for condylar deformity seen on MRI.

In our Pilot Study [30] reduced MOC turned out to be the best associated variable for active TMJ arthritis on MRI. Therefore, our goal was to validate MOC values of JIA patients comparing them with normative values. To our knowledge this is the first study to investigate MOC of JIA patients comparing them with normative values from healthy children, allowing the calculation of age related percentiles for girls and boys separately from 3 through 18 years of age [27]. The mean MOC measured by the rheumatologist and orthodontist were only 0.2 mm and 0.6 mm smaller than in the healthy control group and the average centile was 47.4 and 49.4. Nevertheless, 17 % and 18 % of the patients had a MOC below the 10^th^ percentile during the rheumatologic and the orthodontic examination respectively, and the majority of them showed enhancement on MRI. The overall mean of the MOC measured in our study is in line with a previous investigation [18].

### Clinical parameters associated with TMJ arthritis

In the study of Abramowicz et al. [19], only two clinical findings were significantly associated with synovitis seen on the MRI, the MOC and the deviation on mouth opening. Patients with limited MOC were 6.7 times more likely to have synovitis and therefore the authors concluded that MOC can be used to pre-estimate the presence of TMJ arthritis. Stoll et al. [18] also showed a strong association between mouth-opening deviation and TMJ arthritis in their large cohort of 187 patients. Moreover, a smaller maximal incisal opening and shorter disease duration were associated with an increased risk of TMJ arthritis. In our study a highly significant association between the MOC and severe deformity was observed. Furthermore a significant association between the MOC, the corresponding centile as well as the SD-Score and enhancement as an early sign of TMJ arthritis was detected, but was no longer statistically significant once joints with deformity were excluded. Therefore we can conclude that reduced MOC is a sign of deformation and hence reduced MOC cannot serve as a reliable associated variable for early TMJ arthritis.

In the study of Twilt et al. [5] pain during jaw excursion, absence of translation, asymmetry during maximal opening and protrusion as well as crepitation of the TMJ were recognised to be associated variables for TMJ involvement with a good specificity, but a low sensitivity. In contrast to the result above, Billiau et al. [2] showed no correlation between condylar damage and any clinical findings. In addition, it needs to be mentioned that the first two studies [18, 19] used MRI as their diagnostic gold standard whereas the following two [2, 5] based their correlation on OPT radiographs, a method only able to detect arthritis, when advanced stage bony lesions have already occurred.

The two other clinical findings showing a correlation with the MRI findings—namely antegonial notching and asymmetry—are signs of impaired growth also indicating an already occurred structural damage. Therefore, the only clinical finding with a significant correlation with enhancement is restriction in condylar translation which has already been shown in two previous studies [5, 34]. But in our cohort, restriction in condylar translation lost its significant correlation to MRI enhancement after eliminating all the patients with deformity.

### Limitations

Although this study is the largest so far comparing clincal findings to contrast enhanced TMJ-MRI, it is still limited by the number of patients. A larger cohort might allow for more clinical findings to reach significance to pre-estimate early TMJ arthritis. Furthermore a higher and more balanced representation of JIA subgroups would allow a subgroup-specific analysis revealing a possible difference between the groups. Another limitation is the generously chosen time interval between the MRI and the orthodontic or rheumatological examination of-5.03 days (median −1, SD 20.2 range (−84, 64)) and −23.0 days (median −24, SD 30.5; range (−88, 52)) respectively. However, we assume that this time interval did not severely affect our results because orthodontic and rheumatological examinations showed almost the same results despite most orthodontic examinations beeing scheduled very close to MRI (mean 11.2 days).

## Conclusions

Clinical findings in affected TMJs are correlated with structural damage only. Therefore clinical assessment of TMJs does not allow to diagnose early arthritis accurately and will still depend on contrast enhanced MRI.

## Abbreviations

MOC: Maximal mouth opening capacity
TMJ: Temporomandibular joint
JIA: Juvenile idiopathic arthritis
MRI: Magnetic resonance imaging
SDS: Standard deviation score
